# Transcranial Direct Current Stimulation on the Left Superior Temporal Sulcus Improves Social Cognition in Schizophrenia: An Open-Label Study

**DOI:** 10.3389/fpsyt.2022.862814

**Published:** 2022-06-20

**Authors:** Yuji Yamada, Kazuki Sueyoshi, Yuma Yokoi, Takuma Inagawa, Naotsugu Hirabayashi, Hideki Oi, Aya Shirama, Tomiki Sumiyoshi

**Affiliations:** ^1^Department of Psychiatry, National Center Hospital, National Center of Neurology and Psychiatry, Tokyo, Japan; ^2^Department of Preventive Intervention for Psychiatric Disorders, National Institute of Mental Health, National Center of Neurology and Psychiatry, Tokyo, Japan; ^3^Department of Clinical Data Science, Clinical Research & Education Promotion Division, National Center of Neurology and Psychiatry, Tokyo, Japan

**Keywords:** neuromodulation, transcranial direct current stimulation (tDCS), schizophrenia, social cognition, superior temporal sulcus

## Abstract

**Background:**

Patients with schizophrenia show impairments of social cognition, which cause poor real-world functional outcomes. Transcranial direct current stimulation (tDCS) delivered to frontal brain areas has been shown to partially alleviate disturbances of social cognition. In this study, we aimed to determine whether multisession tDCS targeting the superior temporal sulcus (STS), a brain region closely related to social cognition, would improve social cognitive performance in patients with schizophrenia.

**Methods:**

This was an open-label, single-arm trial to investigate the benefits and safety of multisession tDCS over the left STS. Fifteen patients received tDCS (2 mA × 20 min) two times per day for 5 consecutive days. Anodal and cathodal electrodes were placed over the left STS and right supraorbital regions, respectively. Assessments with the Social Cognition Screening Questionnaire (SCSQ), the Hinting Task (HT), the Brief Assessment of Cognition in Schizophrenia (BACS), and the Positive and Negative Syndrome Scale (PANSS) were conducted at baseline and 1 month after the final stimulation.

**Results:**

Significant improvements were found on theory of mind, as measured using the SCSQ (*d* = 0.53) and the HT (*d* = 0.49). These changes on social cognition were not correlated with those of neurocognition, as measured using the BACS or psychotic symptoms, as measured using the PANSS. There were no adverse events of serious/moderate levels attributable to tDCS.

**Conclusion:**

These results suggest that administration of multisession tDCS with anode stimulation targeting the left STS provides a novel strategy to improve functional outcomes in patients with schizophrenia.

**Ethics Statement:**

The National Center of Neurology and Psychiatry Clinical Research Review Board (CRB3180006) approved this study.

**Trial Registration:**

This study was registered within the Japan Registry of Clinical Trials (jRCTs032180026).

## Introduction

Schizophrenia is one of the most common psychiatric diseases affecting 0.7% of the world population (1). People who develop schizophrenia experience positive (e.g., hallucinations, delusions) and negative (e.g., including apathy, anhedonia, and social withdrawal) symptoms, as well as cognitive impairments, which mostly persist throughout life, if not treated properly (2). During this process, social function often deteriorates (2, 3), causing an unemployment rate of 70% in chronic patients (4).

Cognitive dysfunction is one of the core symptoms of schizophrenia and presents from the early to chronic phases of the illness (3, 5). Impairments of cognitive functions, including neurocognition, social cognition, and metacognition, may be present before the onset of psychosis, become pronounced in the first episode, and continue throughout the entire course of the illness (3). With regard to the treatment of schizophrenia, antipsychotic drugs are used, e.g., to ameliorate positive symptoms, while cognitive dysfunctions are mostly resistant.

Among several types of cognitive function, neurocognition, including learning memory, working memory, executive function, verbal fluency, and attention/information processing, is impaired in schizophrenia (5). Similarly, social cognition, i.e., mental operations underlying social behavior, is also affected (6, 7). It includes emotion recognition, theory of mind (ToM), social perception, and attributional bias (8), whose neural basis may be partially different from that of neurocognition (9). Improvements in social cognition may be directly related to improvements of social functioning (e.g., employment) and the link between neurocognition and social functioning may be mediated by social cognitive functioning (10). Specifically, disturbances of social cognition, including ToM, have been shown to worsen the ability to perform well in occupation and interpersonal relationships in patients with schizophrenia (7, 8, 10). Therefore, the development of therapeutics for impaired social cognition has been intensively pursued in the field of psychiatry (3).

To overcome social cognitive disturbances of schizophrenia, psychosocial (e.g., cognitive rehabilitation) (11) and pharmacological (e.g., second-generation antipsychotic drugs) (12) approaches have been attempted with limited success. As an alternative approach, some types of neuromodulations, particularly non-invasive brain stimulation, e.g., transcranial direct current stimulation (tDCS), have been drawing attention (13). tDCS modulates neural activities in the brain by delivering low-amplitude (usually no more than 2 mA) electrical currents over a short period (generally no more than 30 min) between electrodes, i.e., anode and cathode (14). In a meta-analysis (15), the ability of tDCS on the prefrontal cortex, e.g., the dorsolateral prefrontal cortex (DLPFC), to improve working memory has been shown in patients with schizophrenia (Hedges' g = 0.49). Likewise, it is speculated that tDCS may partially alleviate social cognition impairments that are resistant to antipsychotic drugs (16).

Potential benefits of tDCS for social cognition have been tested in patients with schizophrenia with limited success (13). In a systematic review (13), we reported that anode stimulation on frontal brain areas shows minimal effects on social cognition in these patients. In that report, three articles on tDCS met the inclusion criteria (see Table 1). All the studies adopted 2-mA current with 20-min duration of stimulation. Three studies used single-session online protocols, while one used a two-session online protocol. For outcome measures of social cognition, the following tests have been used: the Awareness of Social Inference Test for ToM, the Bell Lysaker Emotion Recognition Task, and the Mayer–Salovey–Caruso Emotional Intelligence Test for emotion recognition. Social perception was evaluated in one study (19), which used the Profile of Non-verbal Sensitivity (PONS).

**Table 1:** Transcranial direct current stimulation and social cognition impairments in schizophrenia [adapted from Yamada et al. (13)].

**Study**	**Diagnosis**	**Sample size**	**Montage**	**Intensity**	**Duration**	**No. of**	**Evaluation**	**Outcomes**	**Results**
		**(active/sham)**	**(anode/cathode)**	**(mA)**	**(min)**	**sessions**			
Klein et al. (17)	Schizophrenia	36/36	Between P6 and CP6/Left bicep	2	20	1	Online	ER40, BLERT, TASIT	No significant effect
		33/33	AFz/Opposite side of the skull, 1 cm below Iz	2	20	1	Online	ER40, BLERT, TASIT	Limited effects in theory of mind
Rassovsky et al. (18)	Schizophrenia	37/37	F3/Fp2	2	20	2	Online	MSCEIT, TASIT, EIT, EAT	No significant effect
Rassovsky et al. (19)	Schizophrenia	12/12	Fp1/Fp2	2	20	1	Online	MSCEIT, TASIT, PONS, FEIT	Significant effects in emotion recognition

*tDCS, transcranial direct current stimulation; ER40, Emotion Recognition-40; BLERT, Bell Lysaker Emotion Recognition Task; TASIT, The Awareness of Social Inference Test; MSCEIT, Mayer–Salovey–Caruso Emotional Intelligence Test; EIT, Emotion Identification Test; EAT, Empathic Accuracy Task; PONS, Profile of Non-verbal Sensitivity; FEIT, Facial Emotion Identification Test. Each study used the same parameters among active and sham groups. “Limited effects in theory of mind” meant that the exploratory analysis suggested an improvement only in one sub-domain of theory of mind. “Significant effects in emotion recognition” meant that stimulation enhanced the ability to identify facial emotion based on photographs or videos*.

The above studies used 4 patterns of tDCS montage, with the anode/cathode placement on F3/Fp2 (18), Fp1/Fp2 (19), between P6 and CP6/left bicep (17), or AFz/opposite side of the skull, 1 cm below Iz (17) based on the International 10–20 electroencephalography system (Table 1). Frontal brain areas have been adopted for the placement of the anode electrode (17–19), but these studies observed minimal effects on ToM (17) and emotion recognition (18, 19). One study placed anodal electrodes on the right temporoparietal junction (rTPJ), which did not produce positive findings (17). Overall, anodal stimulation of the frontal brain areas or rTPJ has been associated with the lack of benefits for social cognition, suggesting the need to search for effective stimulation sites (13, 20).

To identify the optimal conditions to maximize the benefits of tDCS, it is important to be aware that the neural bases of social cognition and neurocognition are partly different (9, 20). Most studies reporting positive results on neurocognitive function have used the DLPFC for anodal stimulation (15). As noted above, three studies have been conducted to determine the effect of tDCS targeting frontal cortical areas on social cognitive disturbances of schizophrenia (see Table 1) (13, 17–19). Among them, only one study reported facilitative effects of tDCS on emotion recognition with the Fp1 as the anodal site (19). Unlike the case for neurocognition, the neural network for social cognition may include the orbitofrontal cortex, medial prefrontal cortex (mPFC), superior temporal sulcus (STS), and amygdala; functional connectivity of these brain regions is attenuated in schizophrenia (20–22). Accordingly, impaired connectivity between these brain regions is associated with poor social cognitive functioning (6, 9). Especially, the ventral and orbital parts of the mPFC have extensive and reciprocal connections with the limbic system, including the amygdala and surrounding prefrontal regions. Impaired functional connectivity has been indicated for these brain areas in patients with schizophrenia (22).

Among these brain regions, the STS is considered to play a pivotal role in multiple domains of social cognition (Table 2) (20). Furthermore, as tDCS provides electrical currents via the skull, surface areas of the brain, such as STS, provide a feasible target for anodal stimulation. In fact, reduced gray matter volume in the STS was reported to be correlated with severity of social cognitive impairments in patients with schizophrenia (23). On the other hand, stimulation of other brain sites governing social cognition, i.e., the orbitofrontal cortex and mPFC, has been found ineffective (Table 1). Therefore, it was hypothesized that stimulation of the STS would be advantageous for enhancing social cognition.

**Table 2:** Neural basis of social cognition [Yamada et al. (20)].

**Domains of social cognition**	**Corresponding brain regions**
Theory of mind (ToM)	Superior temporal sulcus, Medial prefrontal cortex, Middle temporal gyrus, etc.
Attributional bias	Orbitofrontal cortex, Superior temporal sulcus, Insular cortex, Striatum, Amygdala, etc.
Emotion recognition	Amygdala, Medial prefrontal cortex, Inferior occipital gyrus, Superior temporal sulcus, etc.

These considerations prompted us to determine whether stimulation of skull surface above the STS, e.g., T3 or T4 (mid-temporal), would ameliorate social cognition disturbances in patients with schizophrenia. Since there is little information on the safety of anodal stimulation on temporal brain areas, unlike the case for frontal cortical regions, e.g., F3 (frontal) or Fp1 (front polar), which has been the main target for tDCS (24), this study also investigated the presence/absence of adverse events related to this stimulation method. This constituted a rationale for adopting the present study design, which we have reported elsewhere (20). To the best of our knowledge, this is the first attempt to determine whether tDCS delivered to the skull surface for the STS would enhance social cognitive functioning in patients with schizophrenia.

## Materials and Methods

### Trial Design

This was a single-center trial at the National Center of Neurology and Psychiatry, Tokyo, Japan. An open-label, single-arm study was conducted on 15 participants with a diagnosis of schizophrenia based on the Diagnostic and Statistical Manual of Mental Disorders-5 (DSM-5). We selected an open-label, single-arm design because there was no precedent for tDCS over the left STS and one of the major focuses of this study was to verify the tolerability and safety of tDCS over the STS. This study design was in accordance with the 2013 Standard Protocol Items: Recommendations for Interventional Trials (SPIRIT) Statement (20, 25), and was registered within the Japan Registry of Clinical Trials (Trial ID: jRCTs032180026).

### Participants

Outpatients treated at National Center Hospital, National Center of Neurology and Psychiatry were enrolled. Participants were recruited by referrals from treating psychiatrists. After providing a written informed consent, subjects were screened by a treating psychiatrist to establish whether they met the eligibility criteria.

### Inclusion and Exclusion Criteria

Participants met the following inclusion criteria (20):

(1) Diagnosis of schizophrenia in the DSM-5 made by well-trained and experienced clinicians with extensive clinical research experience in the National Center of Neurology and Psychiatry.

(2) Aged between 20 and 70 years.

(3) Being able to understand the objectives and content of this study and provide consent to participate in it [the ability to consent to participate in this study is considered insufficient, when patients' intelligence quotient (IQ) is <70 or they present with acute psychiatric symptoms].

(4) Having the Social Cognition Screening Questionnaire (SCSQ) scores of <34 points.

Patients with any of the following conditions were excluded from this study:

(1) Present or past history of severe organic lesions in the brain, dementia, or epilepsy.

(2) With alcohol or substance use disorder that was present within 12 months from screening.

(3) Contraindicated against electroconvulsive therapy or tDCS, e.g., severe cardiovascular diseases, such as myocardial infarction, or aneurysms at high risk of rupture.

(4) Were treated with tDCS or other neuromodulations within the past 2 months (we asked whether participants had any history of tDCS or other neuromodulations).

(5) Deemed inappropriate to participate judged by the principal investigator, e.g., when participants' psychiatric symptoms were unstable.

The dose of psychotropic drugs was not changed during this study period. Furthermore, the type and dosage of psychotropic medications were not changed from 8 weeks prior to the baseline assessment.

### Sample Size Calculation

Sample sizes (*n* = 15) were calculated by assuming an estimated mean difference of the UCSD Performance-based Skills Assessment (UPSA-B) scores from baseline to follow-up of 10.6, with a SD of 15.5 (26). In these assumptions, the power of the primary analysis was 0.8, so approximately *n* = 13 was estimated (one-sample Student's *t*-test). Therefore, it was decided to include a total of 15 samples, taking into account the dropouts from the study (20).

### Intervention

Direct current was transmitted through 35 cm^2^ saline-soaked sponge electrodes and the intervention was performed by a 1 × 1 transcranial direct current low-intensity stimulator (Model 1300 A; Soterix Medical Incorporation, New York, USA). The tDCS montage placed the anode in the left STS and the cathode in the contralateral supraorbital region, which corresponded to the T3 (mid-temporal) and FP2 (front polar) regions, respectively. We applied 10 sessions of direct current of 2 mA for 20 min in 5 consecutive days (twice per day, with an interval of 30 min). tDCS was administered by trained psychiatrists or researchers who did not evaluate any outcome measures.

### Outcomes

Patients received psychological and clinical assessments, including the screening evaluation, after being briefed on the purpose of this study and agreeing to participate in it. Clinical data were collected at baseline and 1 month after the final stimulus (see Table 3).

**Table 3:** Study schedule [Yamada et al. (20)].

	**Study period**				
	**Baseline**	**Intervention**			**Follow-up**
Time point	Within 2 weeks before the start of intervention	Day 1	Days 2–4	Day 5	1 Month after the end of the last stimulation
Enrollment
Eligibility screen	X				
Informed consent	X				
Sociodemographic characteristics	X				
Intervention
tDCS (twice/day)		X	X	X	
Assessments
SCSQ	X				X
Hinting task	X				X
FEST	X				X
BACS	X				X
PANSS	X				X
JART	X				
Adverse events	X	X	X	X	X
Prescribed drugs	X	X	X	X	X

*tDCS, transcranial direct current stimulation; SCSQ, Social Cognition Screening Questionnaire; FEST, Facial Emotion Selection Test; BACS, Brief Assessment of Cognition in Schizophrenia; PANSS, Positive and Negative Syndrome Scale; JART, Japanese Adult Reading Test. The timepoint of follow-up evaluation was allowed to be up to 7 days off*.

#### Social Cognition

The primary outcome was scores on the SCSQ (27), which included test of ToM and attributional style. The task comprised 10 short vignettes presenting an interaction between a fictional character and the study participant. Each vignette was read aloud by the tester. The tester then had the subject respond “Yes” or “No” to three questions about the vignette, addressing ToM and attributional style (full score was 40; higher scores indicated better performance).

To evaluate ToM more accurately, we also used the Hinting Task (28). In the Hinting Task, subjects were required to infer real intentions behind indirect speech. The task comprised 10 short passages presenting an interaction between two characters ending with one of the characters uttering a hint. Each passage was read aloud by the tester. The subject was then asked what the character really meant when he/she uttered the hint. If the subject failed to give the correct response, an even more obvious hint was added to the story and the subject was asked again. A correct response was, therefore, scored as 2 or 1, depending on when the response was given (full score was 20; higher scores indicated better performance).

To evaluate emotion recognition, we used the Facial Emotion Selection Test (FEST) (29). The FEST measured the ability to infer emotions from the facial expressions of others. Participants viewed 21 photographs and answered which emotion (joy, sadness, anger, fear, surprise, disgust, or no emotion) it corresponded to. Performance was indexed as the total number of correct answers (full score was 21; higher scores indicated better performance).

#### Neurocognition

The Brief Assessment of Cognition in Schizophrenia (BACS) was used to evaluate cognitive domains that were impaired in patients with schizophrenia, including verbal memory (list learning task), working memory (digit sequencing task), motor speed (token motor task), verbal fluency (verbal fluency task), attention/speed of information processing (symbol coding task), and executive function (Tower of London task). Each of the six measures was standardized by creating z-scores, whereby the mean scores of the healthy participants were set to zero and their SDs were set to one. The higher scores represented better cognition. To provide a standard metric for combining test scores into domains and comparing performance over time, the Brief Assessment of Cognition in Schizophrenia (BACS) scores were converted to *z*-scores, which showed performance relative to healthy people (30). The premorbid IQ was also estimated using the Japanese Adult Reading Test (JART) (31).

#### Psychotic Symptoms

Psychotic symptoms were evaluated by the Positive and Negative Syndrome Scale (PANSS) (32), which consisted of positive syndrome, negative syndrome, and general psychopathology subscales (with scores ranging from 7 to 49, from 7 to 49, and from 16 to 112, respectively). The higher scores indicate the more severe psychotic symptoms.

#### Adverse Events

Adverse events were defined as unwanted experiences seen during tDCS. Serious adverse events were defined as requiring inpatient treatment. Moderate adverse events were defined as requiring therapeutic intervention, and mild adverse events as requiring no therapeutic intervention. The treating physician recorded the symptoms, date of onset, severity, treatment given, and association with study interventions. If symptoms were already present at baseline and did not worsen during tDCS intervention, they were not treated as adverse events. An experienced psychiatrist checked the presence and extent of adverse events and their association with tDCS before and after each session and assessed safety at all the visits during the intervention by a set of questionnaires. Specifically, 13 adverse event items, including headache, neck pain, scalp pain, itching, tingling, burning, scalp redness, sleepiness, fatigue, and nausea, were assessed. The presence/absence, severity (very mild, mild, moderate, or severe), and probable association with the tDCS (no association, little association, possible, probable, or certain) were evaluated for these events. We followed-up any unresolved adverse events after trial completion.

### Data Collection and Management

The assessments were conducted at baseline and 1 month after the end of the last stimulation (Table 3). All evaluations were conducted by experienced psychologists. All the data were recorded to the Electronic Data Capture system (HOPE eACReSS; Fujitsu, Tokyo, Japan), which is a secure system designed for storage of personal and patient data. The data were sent to independent data managers to assess whether the data were collected properly, focusing on the status of consent acquisition, eligibility of participants, evaluation items, and confirmation of dropout/terminated cases. These data managers also oversaw and reviewed the progress of the trial. The Efficacy and Safety Assessment Committee, whose members were independent of the study and came from the National Center of Neurology and Psychiatry, checked and assessed whether the trial was conducted safely and properly, and also decided whether to stop the trial, if any severe adverse events or protocol violations occurred. In addition, an on-site data monitor conducted monitoring to ensure the trial was performed properly, data were properly recorded, and data reliability was ensured. If we conducted any necessary protocol modifications, we reported them to the Clinical Research Review Board and to the Ministry of Health, Labor and Welfare for registration in the Japan Registry of Clinical Trials website (https://jrct.niph.go.jp).

### Statistical Analysis

Normality was considered for baseline and change values of demographic (i.e., age, gender, and duration of disease) and clinical outcome (i.e., SCSQ, Hinting Task, FEST, BACS, and PANSS) parameters. Since the sample size of this study is relatively small, we checked distribution of each variable by examining histograms to see, if there is any trend to doubt the normality of collected data.

For evaluating efficacy variables, the Student's paired *t*-test was used for the clinical outcomes and their effect sizes were calculated as standardized mean difference (Cohen's d). Correlations between baseline values and change of scores on clinical outcomes were evaluated by single regression analysis. Safety data were collected by a common questionnaire and were not subjected to statistical analysis. Statistical analysis was conducted using STATA version 15, created by Stata Corporation in Texas, USA.

## Results

### Participants

Fifteen outpatients were enrolled and completed this study without any dropout. Baseline characteristics of patients are shown in Table 4. No medication was changed during this study period and cognitive rehabilitation was not performed for the participants.

**Table 4:** Clinical characteristics of patients (*n* = 15).

**Variables**	**Mean (SD) or *n***
Inpatient/outpatient	0/15
Male/female	7/8
Age (year)	40.1 (11.8)
Married/unmarried	3/12
Living alone/living with family	5/10
Employed/unemployed	3/12
Duration of education (year)	13.6 (1.8)
Premorbid IQ	102.1 (11.0)
Duration of present illness (year)	12.6 (10.2)
Number of past hospitalizations	3.0 (3.1)
Chlorpromazine equivalent dose of antipsychotics (mg/day)	727.9 (323.1)[min. 300, max. 1,400]
Diazepam equivalent dose of benzodiazepines (mg/day)	14.2 (14.6)[min. 0, max. 40]
Imipramine equivalent dose of antidepressants (mg/day)	21.2 (59.2)[min. 0, max. 225]

*SD, standard deviation; IQ, Intelligence Quotient. Equivalent doses of chlorpromazine, diazepam, and imipramine were calculated (33)*.

### Effect of tDCS on Cognition

Significant improvements were found on the SCSQ, and the Hinting Task scores. Improvements of the SCSQ and the Hinting Task were associated with medium effect sizes. On the other hand, no significant change was found on FEST or BACS scores (Table 5).

**Table 5:** Outcome measures at baseline and 1 month after the tDCS.

	**Baseline, mean (SD)** **[range]**	**Follow-up, mean (SD)** **[range]**	***t*-value** **(degree of freedom)** **or *z*-Value**	***p*-value**	**Effect size**
**SCSQ**					
Total	29.93 (4.49) [20.33–33.66]	32.17 (3.91) [26.00–38.00]	*t* = −2.356 (14)	**0.034**	***d*** **= 0.53**
**Hinting task**					
Total	15.33 (3.51) [8.00–20.00]	16.93 (3.03) [11.00–20.00]	*t* = −2.449 (14)	**0.028**	***d*** **= 0.49**
**FEST**					
Total	13.20 (2.51) [8.00–17.00]	13.46 (2.85) [8.00–17.00]	*t* = −0.718 (14)	0.484	*d* = 0.10
**BACS (** ***z*** **-score)**					
Composite score	−1.85 (1.41) [−4.14–0.67]	−1.79 (1.33) [−4.46–0.55]	*z* = −0.469	0.646	*d* = 0.04
**PANSS**					
Positive syndrome	16.20 (6.01) [8.00–27.00]	15.60 (6.34) [8.00–31.00]	*t* = 0.788 (14)	0.444	*d* = 0.10
Negative syndrome	19.33 (6.47) [11.00–31.00]	17.93 (6.29) [8.00–28.00]	*t* = 1.567 (14)	0.139	*d* = 0.22
General psychopathology	37.53 (9.28) [25.00–54.00]	33.46 (9.20) [21.00–52.00]	*t* = 4.077 (14)	**0.001**	***d*** **= 0.44**

*tDCS, transcranial direct current stimulation; SD, standard deviation; SCSQ, Social Cognition Screening Questionnaire; FEST, Facial Emotion Selection Test; BACS, Brief Assessment of Cognition in Schizophrenia; PANSS, Positive and Negative Syndrome Scale. Values reaching statistical significance are bolded*.

### Psychotic Symptoms

Improvement was found on the PANSS general psychopathology subscale scores with a small effect size. On the other hand, no significant improvement was found for positive syndrome and negative syndrome subscale scores (Table 5).

### Correlations Between Psychotic Symptoms and Cognitive Data

No correlations were found between the PANSS positive syndrome, negative syndrome, and general psychopathology scores at baseline and changes in scores on the SCSQ (*r* = −0.154, *p* = 0.366; *r* = −0.040, *p* = 0.803; *r* = −0.083, *p* = 0.453, respectively), the Hinting Task (*r* = −0.108, *p* = 0.355; *r* = 0.121, *p* = 0.262; *r* = 0.029, *p* = 0.704, respectively), and the FEST (*r* = −0.099, *p* = 0.124; *r* = 0.001, *p* = 0.986; *r* = 0.077, *p* = 0.059, respectively). Likewise, changes in the PANSS positive syndrome, negative syndrome, or general psychopathology scores were not correlated significantly with changes in scores on the SCSQ (*r* = −0.086, *p* = 0.701; *r* = −0.265, *p* = 0.307; *r* = −0.403, *p* = 0.157, respectively), Hinting Task (*r* = 0.373, *p* = 0.245; *r* = −0.083, *p* = 0.831; *r* = 0.040, *p* = 0.926, respectively), or FEST (*r* = 0.567, *p* = 0.318; *r* = −0.014, *p* = 0.984; *r* = −1.028, *p* = 0.159, respectively).

### Adverse Events

Some mild or moderate adverse events were recorded, e.g., itching (60%), sleepiness (46.7%), and tingling (20%). However, two cases with adverse events of the “Moderate” severity turned out not requiring specific medical treatments, and should have been reported as “Mild”. On the other hand, serious adverse events were not observed (Table 6).

**Table 6:** Adverse events related to tDCS reported by patients.

**Adverse Events**	***N* (%)**	**Severity of adverse events (*N*)**	**Intensity of association (*N*)**
Headache	1 (6.6%)	Moderate (1)	Possible (1)
Neck pain	2 (13.3%)	Very mild (1), Mild (1)	Little association (2)
Scalp pain	0 (0%)	–	–
Tingling	3 (20%)	Very mild (2), Mild (1)	Little association (1), Possible (2)
Itching	9 (60%)	Very mild (8), Mild (1)	Little association (1), Possible (8)
Burning sensation	0 (0%)	–	–
Skin redness	0 (0%)	–	–
Sleepiness	7 (46.7%)	Very mild (6), Mild (1)	No association (1), Little association (3), Possible (3)
Trouble concentrating	0 (0%)	–	–
Tiredness	1 (6.6%)	Very mild (1)	Little association (1)
Dizziness	1 (6.6%)	Moderate (1)	Possible (1)
Nausea	1 (6.6%)	Very mild (1)	Little association (1)
Others	0 (0%)	–	–

*N, number; tDCS, transcranial direct current stimulation. Severity of adverse events was rated on a scale of 4, i.e., very mild, mild, moderate, or severe. Intensity of association was rated on a scale of 5, i.e., no association, little association, possible, probable, or certain. These cases with severity of “Moderate” turned out not requiring medical treatments*.

## Discussion

To the best of our knowledge, this study is the first to suggest the ability of multisession tDCS delivered to the left STS to improve social cognition, especially ToM, in patients with schizophrenia. These effects of tDCS were not associated with changes on neurocognition or psychosis.

So far, three studies have been conducted to examine the ability of tDCS to improve social cognitive disturbances of schizophrenia (see Table 1) (13, 17–19). Among them, only one study reported facilitative effects of stimulation on the left prefrontal cortex on emotion recognition (19). However, there has been little evidence that tDCS, with anodes on frontal brain regions, improves ToM, a core domain of social cognition, suggesting the need for alternative regimens. It should be noted that the current study is an open-label trial, whereas the previous study with a large sample size (17–19) used randomized controlled designs. Further trials with a more rigorous design are essential to confirm the efficacy of tDCS delivered to the STS for ameliorating social cognitive disturbances of schizophrenia.

The neural circuit underlying social cognition involves the left STS, corresponding to T3 (mid-temporal). Accordingly, it was hypothesized that anodal tDCS targeting this brain region would be effective in alleviating social cognitive disturbances in patients with schizophrenia (Table 2) (13, 20, 34). As expected, anodal stimulation of the left STS was found to improve performance on the SCSQ and the Hinting Task, tests of social cognition, especially ToM, with medium effect sizes. The lack of correlation between changes on the SCSQ or the Hinting Task scores and severity of psychotic symptoms at baseline or its change suggests that the change of ToM performance is not secondary to amelioration of psychotic symptoms.

To effectively enhance specific domains of symptoms/cognitive function, neural circuits underlying them should be considered. So far, most studies with positive results on neurocognitive function used the DLPFC for anodal stimulation (15). By contrast, stimulation of the same brain region mostly failed to produce benefits for social cognition, e.g., ToM (17, 18). The negative results in previous studies may have been due to the difference in neural circuits governing respective cognitive functions (neurocognition *vs*. social cognition) (9, 20, 22). As neural substrates for social cognition include the orbitofrontal cortex, mPFC, STS, and amygdala (20, 21), we chose the left STS for anodal stimulation. As expected, tDCS delivered to the skull for this brain area was found to improve ToM (measured by the Hinting Task and the SCSQ), but not neurocognition (measured by the BACS). These results suggest that the left STS may provide an optimal stimulation site for alleviating social cognitive impairments of schizophrenia.

Findings of this study indicate multisession tDCS may be advantageous for producing later improvement of social cognition in patients with schizophrenia. The effect sizes of tDCS for this benefit were about 0.5 SD) (Table 5), which is considered clinically meaningful (7). By contrast, its immediate effects after 1–2 sessions tDCS have been reported not to improve social cognitive disturbances (Table 1) (13, 17–19). As social cognition is considered to be directly linked to real-world social functioning (3, 10), multisession tDCS, rather than single-session tDCS, may provide benefits for functional recovery in patients with schizophrenia. Further investigations to elucidate the time course of the effect of multisession tDCS are warranted.

Improvements of cognitive symptoms by multisession tDCS may be mediated through several mechanisms, including long-term potentiation (LTP), continuous enhancement of signal transduction between neurons, and related neural events (14, 34, 35). Specifically, LTP has been shown to enhance the synthesis of various proteins, e.g., neurotransmitter synthases, receptors, ion channels, and intracellular signal proteins, thus facilitating the efficiency of neurotransmissions in cortical circuits (14, 34, 35). Moreover, improvement in cognitive function becomes more apparent a few weeks after administration of tDCS (36), suggesting that tDCS may induce LTP.

As this was the first trial using tDCS to the STS, an open-label, single-arm study design was adopted to ensure safety (20). Although some mild adverse events were observed, serious or moderate adverse events were absent. These findings suggest that the method of tDCS, used here, would be safe and feasible.

The facilitative effect of tDCS on social cognition in patients with schizophrenia may provide some clinical implications. First, the effect sizes obtained in this study are no less than those of cognitive rehabilitation that requires a greater constraint of time for patients and medical staffs (11). Second, effect sizes of tDCS on social cognition, reported here, are also greater than those of pharmacotherapy (37–39), while tDCS is only associated with local insults in most cases, unlike the case for medications that penetrate into the whole body. Therefore, tDCS may be useful in patients who cannot receive other modalities of treatment due to time constraints or intolerance caused by systemic side effects.

## Limitations

The lack of blinding associated with this study design might have produced practice (repeated measure) effect in some measures used. Second, the small sample size may raise caution in concluding that these results represent effects in the population. Third, the lack of randomization, controlled group, and blinding might have produced placebo effects. A randomized controlled trial is currently considered to confirm the benefit of tDCS targeting the left STS for social cognition in schizophrenia and other psychiatric conditions.

## Conclusion

In conclusion, the results of this study suggest that tDCS delivered to the left STS may produce potential benefits for some domains of social cognition, especially ToM, in patients with schizophrenia. These observations provide a novel therapeutic strategy to improve functional outcomes for these patients.

## Data Availability Statement

The raw data belonged to the present study cannot be made publicly available, because the disclosure of personal data was not included in the research protocol.

## Ethics Statement

The protocol for this study was reviewed and approved by National Center of Neurology and Psychiatry Clinical Research Review Board (CRB3180006). The participants provided their written informed consent to participate in this study.

## Author Contributions

YujY developed the original concept for the trial, established the protocol, and wrote the initial version of the manuscript. YujY and TS designed the trial. YumY advised the statistical analysis, while KS advised the outcome measures. AS and YujY administered tDCS. TI, NH, and TS recruited participants. HO managed the data. TS organized the team for this study. All the other authors have reviewed and commented on the subsequent draft, and agreed to the published version of the manuscript.

## Funding

This study was partially supported by the Japan Society for the Promotion of Science KAKENHI Grant No. 20K16635 to YujY, as well as JSPS KAKENHI Grant No. 20H03610, the Intramural Research Grants for Neurological and Psychiatric Disorders of NCNP (2-3, 3-1), and the Japan Health Research Promotion Bureau Grants (2020-B-08, 2021-B-01) to TS.

## Conflict of Interest

The authors declare that the research was conducted in the absence of any commercial or financial relationships that could be construed as a potential conflict of interest.

## Publisher's Note

All claims expressed in this article are solely those of the authors and do not necessarily represent those of their affiliated organizations, or those of the publisher, the editors and the reviewers. Any product that may be evaluated in this article, or claim that may be made by its manufacturer, is not guaranteed or endorsed by the publisher.
